# Radiotherapy-Induced Neuronal Dysfunction in Patients with Brain Tumors: Dose–Volume Effects, Imaging Biomarkers and Clinical Implications

**DOI:** 10.3390/diagnostics16101528

**Published:** 2026-05-18

**Authors:** Carla-Bianca Vulturar, Nicolae Verga, Olivian Savencu, Flonta Teodora

**Affiliations:** 1 Department of Radiation Oncology, Clinic Medeuropa, 410191 Oradea, Romania; 2 Doctoral School of Medicine, “Carol Davila” University of Medicine and Pharmacy, 050474 Bucharest, Romania; 3 Department of Oncological Radiotherapy and Medical Imaging, “Carol Davila” University of Medicine and Pharmacy, 050474 Bucharest, Romania

**Keywords:** brain tumors, radiotherapy, radiation-induced brain injury, neuronal dysfunction, dose–volume effects, neuroimaging, cognitive impairment

## Abstract

**Background:** Brain tumors represent a major cause of neurological morbidity and mortality, often requiring radiotherapy as a central component of treatment. While advances in radiation techniques have improved tumor control, increasing attention has been directed toward radiation-induced effects on healthy brain tissue, particularly regarding neuronal function and cognitive outcomes. **Objective:** This review aims to provide a structured synthesis of current evidence on radiation-induced neuronal dysfunction, integrating dose–volume parameters, neuroimaging biomarkers, and clinical neurological manifestations. **Methods:** A structured literature review was conducted using electronic databases including PubMed, Scopus, and Web of Science. Relevant studies evaluating dose–volume effects, neuroimaging findings, and clinical outcomes following cranial radiotherapy were included. **Results:** Dose–volume histogram (DVH) parameters, including mean brain dose and intermediate-dose volumes (V10–V30), as well as hippocampal dose, were identified as key factors associated with cognitive decline and neuronal dysfunction. Conventional MRI detects structural changes such as white matter injury and radionecrosis, while advanced techniques including diffusion tensor imaging (DTI) and functional MRI (fMRI) reveal microstructural damage and network disruption. These imaging findings correlate with a spectrum of clinical manifestations ranging from subtle cognitive impairment to significant neurological deficits. **Conclusions:** Radiation-induced neuronal dysfunction represents a complex and multifactorial process that extends beyond localized tissue injury. Integrating dose–volume considerations with advanced imaging biomarkers may improve risk stratification and support the development of neuroprotective strategies in patients undergoing cranial radiotherapy.

## 1. Introduction

Brain tumors represent a significant cause of neurological morbidity and mortality worldwide, affecting both structural and functional integrity of the central nervous system. Radiotherapy plays a fundamental role in the multidisciplinary management of both primary and metastatic brain tumors, contributing substantially to local tumor control and overall survival. Continuous advances in neurosurgery, systemic therapies, and radiation delivery techniques have improved survival outcomes, consequently shifting clinical attention toward treatment-related neurological toxicity and long-term preservation of brain function rather than survival alone [1,2].

Despite its therapeutic efficacy, cranial radiotherapy inevitably results in irradiation of surrounding healthy brain tissue. Exposure of normal neural structures to radiation may induce a spectrum of biological alterations collectively referred to as radiation-induced brain injury. These changes may occur across acute, early-delayed, and late phases following treatment and are increasingly recognized as major contributors to neurological deterioration and reduced quality of life among brain tumor survivors [3]. Importantly, radiation-related damage is not restricted to high-dose regions but may also arise from cumulative exposure of large volumes of normal brain tissue to intermediate and low radiation doses, emphasizing the relevance of dose–volume relationships in contemporary radiotherapy planning [4].

At the cellular level, radiation-induced neuronal dysfunction represents a complex and multifactorial process involving oxidative stress, neuroinflammatory activation, vascular endothelial injury, demyelination, and disruption of neuronal stem cell niches. Experimental and clinical studies have demonstrated that cranial irradiation significantly impairs hippocampal neurogenesis, a process essential for learning and memory formation, thereby providing a biological substrate for post-treatment cognitive decline [5,6]. Persistent microglial activation and chronic neuroinflammation further contribute to synaptic dysfunction and progressive white matter injury, ultimately affecting neuronal connectivity and functional brain networks [7].

Advances in neuroimaging have enabled earlier detection and characterization of radiation-induced cerebral alterations. Magnetic resonance imaging (MRI), particularly T2-weighted and FLAIR sequences, frequently demonstrates white matter changes, edema, and treatment-related necrosis following radiotherapy. These imaging biomarkers are increasingly used as surrogate indicators of underlying neuronal and microvascular injury and may correlate with clinical manifestations such as cognitive impairment, seizures, fatigue, and increased dependence on anti-edematous therapies [8,9].

The introduction of highly conformal radiation techniques, including intensity-modulated radiotherapy (IMRT) and volumetric modulated arc therapy (VMAT), has enabled improved sparing of critical neural structures such as the hippocampus and temporal lobes. Clinical evidence suggests that reduction in radiation dose to these radiosensitive regions may mitigate neurocognitive decline without compromising oncological outcomes [10]. Nevertheless, the quantitative relationship between radiation dose distribution, imaging evidence of brain injury, and clinically relevant neurological dysfunction remains incompletely understood.

Beyond tumor control, preservation of neurological integrity has become an essential therapeutic objective in contemporary neuro-oncology. Understanding how radiotherapy influences neuronal function is therefore not only a scientific necessity but also a clinical responsibility toward maintaining patients’ cognitive independence and quality of life. The human brain represents not only the target of oncological treatment but also the biological substrate of cognition, behavior, and personal identity. Consequently, investigating radiation-induced changes in healthy brain tissue becomes fundamental for understanding the true impact of radiotherapy beyond tumor response alone.

The aim of this review is to summarize current evidence regarding the biological mechanisms underlying radiation-induced neuronal injury, the impact of dose–volume parameters on healthy brain tissue, imaging correlates of post-radiotherapy brain alterations, and their clinical implications in patients with brain tumors.

## 2. Methods

### 2.1. Literature Search Strategy

A structured literature search was performed to identify relevant studies addressing radiation-induced neuronal dysfunction, dose–volume relationships, and neuroimaging biomarkers in patients undergoing cranial radiotherapy. Electronic databases including PubMed, Scopus, and Web of Science were systematically searched for articles published between January 2000 and December 2024.

The search strategy combined the following keywords and Medical Subject Headings (MeSH): “radiotherapy”, “brain tumors”, “radiation-induced brain injury”, “neuronal dysfunction”, “cognitive impairment”, “dose–volume effects”, “DVH”, “hippocampus”, “functional MRI”, and “diffusion tensor imaging”.

Only articles published in English were included.

### 2.2. Study Selection Criteria

Studies were included if they:

(i) Evaluated radiation-induced changes in brain structure or function;

(ii) Reported dose–volume parameters or radiotherapy-related dosimetric data;

(iii) Investigated neuroimaging findings or clinical neurological outcomes;

(iv) Included adult patients with primary or metastatic brain tumors.

Exclusion criteria comprised case reports with insufficient data, non-clinical experimental studies lacking translational relevance, and studies without clearly defined radiotherapy parameters.

### 2.3. Data Extraction and Synthesis

Data extracted from the selected studies included radiotherapy technique, dose–volume parameters (e.g., mean dose, V10–V30), affected brain regions, imaging biomarkers, and reported clinical outcomes.

The findings were synthesized qualitatively, with emphasis on identifying consistent relationships between radiation dose distribution, structural and functional brain alterations, and neurological manifestations.

## 3. Biological Mechanisms of Radiation-Induced Neuronal Injury

Radiation-induced injury of the central nervous system represents a multifaceted biological phenomenon extending beyond direct cellular damage. Although mature neurons exhibit limited proliferative activity and were historically considered relatively resistant to radiation exposure, current evidence indicates that neuronal dysfunction primarily results from indirect alterations affecting the complex cellular and vascular microenvironment that sustains normal brain function. Consequently, radiation-induced neurological impairment should be understood as a dynamic process involving interactions between neural, glial, vascular, and inflammatory components rather than isolated neuronal loss alone [11,12].

One of the earliest biological responses to ionizing radiation is the excessive generation of reactive oxygen species and persistent oxidative stress. Radiation-induced DNA damage and mitochondrial disruption initiate molecular cascades that impair cellular metabolism and compromise neuronal homeostasis. Unlike acute radiation effects observed in rapidly dividing tissues, oxidative injury within the brain may persist long after treatment completion, promoting progressive structural and functional alterations even in regions exposed to moderate radiation doses [2,13]. This sustained oxidative imbalance contributes to delayed neuronal vulnerability and may explain the gradual onset of neurological symptoms frequently observed during post-radiotherapy follow-up.

Neuroinflammation has emerged as a central mediator of radiation-induced neuronal dysfunction. Following cranial irradiation, activation of microglia and astrocytes results in prolonged secretion of pro-inflammatory cytokines and chemokines, generating a chronic inflammatory microenvironment within the irradiated brain. Persistent inflammatory signaling interferes with synaptic plasticity, alters neuronal communication pathways, and accelerates degeneration of white matter structures [7,14]. Importantly, experimental studies suggest that functional impairment may develop in the absence of extensive neuronal death, indicating that disruption of neuronal networks rather than cell loss alone plays a critical role in post-irradiation neurological decline [7,14].

Vascular injury represents another fundamental mechanism underlying radiation-related brain damage. Ionizing radiation induces endothelial dysfunction, capillary loss, and increased permeability of the blood–brain barrier. These alterations impair cerebral perfusion and promote tissue hypoxia, facilitating edema formation and secondary neuronal injury [15]. From a clinical perspective, such mechanisms are frequently reflected by radiological findings including T2/FLAIR hyperintensities and treatment-related necrosis observed during imaging surveillance after radiotherapy.

Particular attention has been directed toward radiation effects on neurogenic niches, especially within the hippocampal dentate gyrus and the subventricular zone. Neural stem and progenitor cells located in these regions demonstrate marked radiosensitivity, and even relatively low radiation doses may significantly suppress neurogenesis [5,6]. Given the essential role of hippocampal neurogenesis in memory consolidation and learning processes, impairment of these regenerative mechanisms provides a biologically plausible explanation for cognitive alterations described in patients receiving cranial irradiation.

Beyond gray matter injury, radiation-induced damage to white matter structures substantially contributes to neurological dysfunction. Oligodendrocyte precursor cells are highly susceptible to radiation exposure, and their depletion leads to demyelination and impaired axonal signal transmission. Progressive disruption of myelin integrity compromises large-scale neuronal connectivity networks responsible for executive function, attention, and information processing speed [16]. Such alterations may precede macroscopic imaging abnormalities, suggesting that functional network disruption represents an early manifestation of radiation injury.

Emerging data further indicate that synaptic architecture itself may be affected by radiation exposure. Experimental observations have demonstrated reductions in dendritic spine density and alterations in neurotransmission following irradiation, ultimately disturbing neuronal network synchronization essential for cognitive performance [2,17]. These findings reinforce the concept that radiation-induced neuronal dysfunction frequently reflects impaired connectivity rather than overt neuronal destruction.

Mitochondrial dysfunction also contributes significantly to delayed radiation toxicity. Disruption of cellular energy metabolism results in reduced adenosine triphosphate production and increased susceptibility of metabolically active neuronal populations to oxidative injury [18]. Regions characterized by high energetic demand, including cortical and hippocampal structures, therefore appear particularly vulnerable to cumulative radiation effects.

Increasing attention has also been directed toward radiation-induced disruption of the neurovascular unit, an integrated system composed of neurons, endothelial cells, astrocytes, and pericytes responsible for maintaining cerebral homeostasis. Damage to this regulatory interface leads to chronic blood–brain barrier instability and sustained inflammatory activation, promoting long-term tissue injury and persistent edema formation [19]. These mechanisms contribute to the radiological and clinical heterogeneity frequently encountered after cranial radiotherapy.

Importantly, radiation-induced brain injury evolves over time. Acute effects occurring during treatment are commonly associated with transient inflammatory responses, whereas early delayed injury reflects demyelination and reversible white matter changes. Late radiation toxicity, which may develop months or years after therapy, is primarily characterized by vascular compromise, necrosis, and irreversible neuronal dysfunction [3,16]. Recognition of this temporal continuum is essential for accurate interpretation of post-treatment imaging findings and clinical symptom evolution.

Individual susceptibility to radiation-induced neuronal injury varies considerably among patients and depends on multiple interacting factors, including total radiation dose, fractionation scheme, irradiated brain volume, patient age, and concomitant systemic therapies [2]. Increasing evidence suggests that biological variability and inflammatory responses may further influence treatment tolerance, supporting the need for personalized radiotherapy strategies focused on functional brain preservation.

Taken together, radiation-induced neuronal dysfunction results from cumulative interactions between oxidative stress, neuroinflammation, vascular injury, impaired neurogenesis, and disruption of neuronal connectivity. Understanding these interconnected mechanisms provides the biological framework necessary for interpreting imaging biomarkers and clinical manifestations observed following cranial irradiation and supports ongoing efforts aimed at optimizing radiotherapy delivery while preserving neurological function [20].

## 4. Dose–Volume Effects on Healthy Brain Tissue

Understanding the relationship between radiation dose distribution and normal brain tissue response represents a fundamental aspect of contemporary cranial radiotherapy. While tumor control remains the primary therapeutic objective, increasing attention has been directed toward the unintended exposure of surrounding healthy cerebral structures and its potential functional consequences. Modern treatment planning has demonstrated that neurological toxicity is not exclusively related to maximum delivered dose but rather reflects complex interactions between total dose, irradiated volume, and regional brain sensitivity.

The concept of dose–volume effects has therefore become central in evaluating radiation-induced brain injury. Parameters derived from dose–volume histograms (DVH), including mean dose and volume-based thresholds, provide quantitative measures of radiation exposure to normal brain tissue. Clinical and experimental evidence suggests that even moderate radiation doses delivered to large volumes of healthy brain parenchyma may contribute to progressive functional impairment, particularly in long-term survivors [3].

Certain cerebral regions demonstrate increased susceptibility to radiation exposure due to their structural organization and functional specialization. Among these, the hippocampus has received considerable attention because of its essential role in memory processing and neurogenesis. Studies evaluating hippocampal dose constraints have shown that preservation of this structure during cranial irradiation may reduce the risk of post-treatment cognitive decline, supporting the concept that selective sparing of critical neural substrates can influence neurological outcomes without compromising treatment efficacy [10].

Clinical and imaging studies have further demonstrated that increased radiation dose to the hippocampus is associated with measurable cognitive decline, particularly in memory function. Dose–volume parameters, including mean hippocampal dose, have been identified as significant predictors of neurocognitive outcomes, supporting the concept that hippocampal sparing may reduce treatment-related toxicity [21,22].

These dose–volume relationships and their associated neurological outcomes are summarized in Table 1.

From a clinical perspective, these findings underscore the importance of incorporating dose–volume considerations into radiotherapy planning to minimize exposure of functionally critical brain regions. In particular, limiting mean brain dose and reducing intermediate-dose exposure (V10–V30) to large brain volumes may help mitigate the risk of cognitive decline. Preservation of the hippocampus has emerged as a key strategy, given its central role in memory function and demonstrated sensitivity to radiation.

These observations support a shift from purely target-oriented radiotherapy toward function-preserving approaches, in which treatment planning balances tumor control with the protection of neural networks underlying cognitive and neurological function. Such strategies may contribute to improved long-term outcomes and quality of life in patients undergoing cranial irradiation.

Beyond focal structures, the cumulative irradiation of normal brain tissue outside the target volume—commonly referred to as *brain minus planning target volume (brain–PTV)*—has emerged as an important determinant of treatment-related toxicity. Exposure of extensive white matter regions to intermediate radiation doses may promote microvascular injury, demyelination, and chronic inflammatory responses, mechanisms that are frequently reflected by diffuse imaging abnormalities observed during follow-up examinations. These findings suggest that neurological effects may arise not only from localized high-dose regions but also from widespread low- and intermediate-dose exposure.

The spatial distribution of radiation dose also appears to influence post-treatment cerebral response. Peritumoral brain tissue represents a particularly vulnerable region, as it is frequently affected by pre-existing edema, surgical manipulation, and altered vascular integrity prior to irradiation. Additional radiation exposure in these areas may exacerbate blood–brain barrier disruption and inflammatory activation, contributing to persistent edema and delayed tissue recovery. Clinically, such processes may manifest through prolonged dependence on corticosteroid therapy or the need for intensified anti-edematous management.

Importantly, dose–volume relationships should be interpreted within the broader clinical context. Patient-related factors, including age, baseline neurological status, tumor location, and concomitant systemic therapies, may significantly modify individual tolerance to radiation exposure. Furthermore, advances in conformal radiotherapy techniques have demonstrated that improved dose shaping allows meaningful reduction in radiation delivered to functionally relevant brain regions, reinforcing the principle that treatment planning decisions directly influence neurological outcomes.

Recent developments in treatment optimization increasingly emphasize functional preservation alongside oncological effectiveness. Rather than considering normal brain tissue as a homogeneous organ, contemporary radiotherapy approaches recognize regional variability in radiosensitivity and functional importance. This paradigm shift supports individualized treatment planning strategies aimed at minimizing radiation exposure to critical neural networks while maintaining adequate target coverage.

An additional aspect that remains insufficiently emphasized in current literature is the potential functional relevance of spatially heterogeneous dose distribution within the healthy brain. Even when global dose constraints are respected, small regions receiving intermediate radiation doses may overlap with functionally interconnected neural networks rather than isolated anatomical structures. Consequently, radiation exposure may disrupt network-level brain organization without producing immediately detectable focal injury. This concept suggests that radiation-induced neurological impairment may reflect alterations in large-scale functional connectivity rather than localized tissue damage alone, offering a possible explanation for discrepancies frequently observed between imaging findings and clinical symptom severity.

Furthermore, clinical experience indicates that treatment-related neurological deterioration is often multifactorial, arising from the cumulative interaction between pre-existing tumor-related alterations and radiation exposure. In this context, radiotherapy may act not solely as an injurious factor but as a physiological stressor superimposed on an already vulnerable neural environment. Recognizing this interaction may contribute to a more nuanced interpretation of post-treatment imaging changes and supports the growing need for individualized assessment of normal brain tolerance.

**Figure 1:**
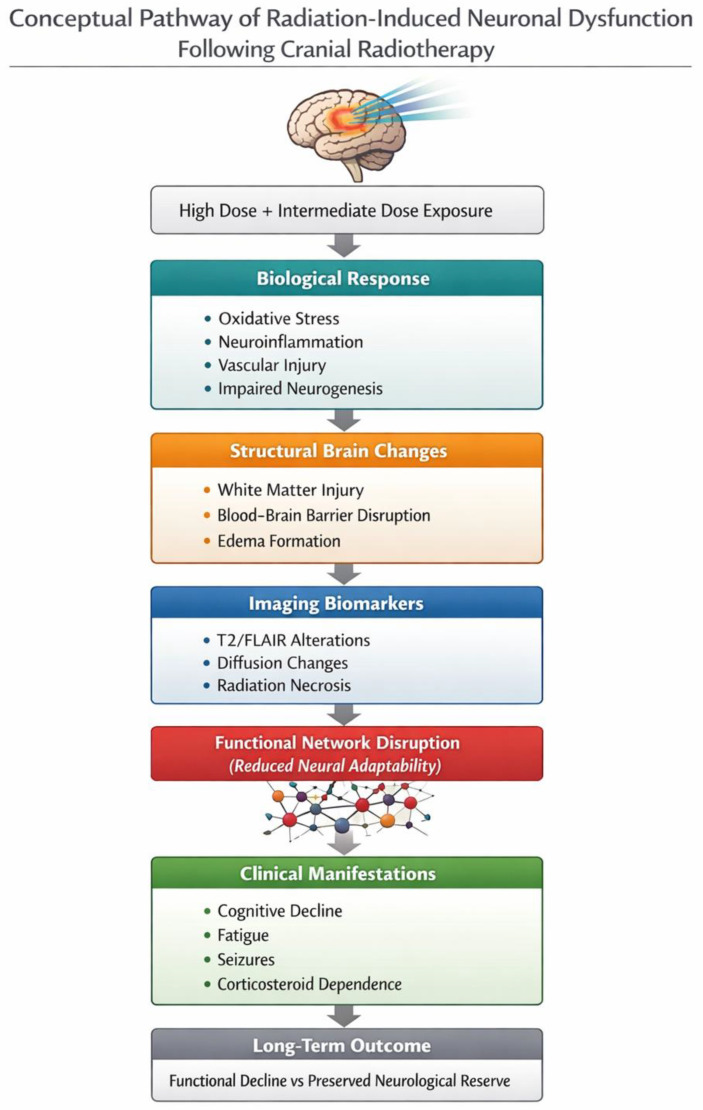
Conceptual illustration of the biological and clinical cascade underlying radiation-induced neuronal dysfunction following cranial radiotherapy. Radiation dose distribution initiates molecular and vascular responses leading to structural brain alterations, imaging biomarkers, and clinical neurological manifestations. *Schematic illustration created by the authors for educational purposes.*

Taken together, current evidence indicates that radiation-induced neuronal dysfunction reflects not only total administered dose but also the volume and anatomical distribution of irradiated healthy brain tissue. A comprehensive understanding of dose–volume effects therefore represents an essential step toward balancing tumor control with preservation of neurological function in patients undergoing cranial radiotherapy, as illustrated in Figure 1.

## 5. Imaging Biomarkers of Radiation-Induced Brain Injury

Neuroimaging represents one of the few interfaces through which clinicians can observe the biological consequences of radiotherapy within the living brain. While imaging has traditionally been employed to evaluate tumor response, its role has progressively expanded toward understanding treatment-induced alterations affecting normal cerebral tissue. In this context, magnetic resonance imaging (MRI) functions not merely as a diagnostic modality but as a window into the dynamic interaction between radiation exposure and neuronal integrity.

Radiation-induced brain injury rarely manifests as an abrupt structural event. Instead, imaging changes often resemble a gradual reorganization of cerebral tissue, reflecting cumulative biological stress rather than discrete damage. T2-weighted and fluid-attenuated inversion recovery (FLAIR) sequences frequently demonstrate diffuse white matter hyperintensities following cranial irradiation, findings that may initially appear disproportionate to the patient’s clinical presentation [23,24]. Much like fatigue developing after prolonged systemic illness rather than acute trauma, these radiological alterations evolve progressively, mirroring underlying inflammatory and microvascular processes.

A recurring clinical observation raises an important question: why do some patients exhibit significant imaging abnormalities with minimal symptoms, whereas others develop pronounced neurological deterioration despite relatively modest radiological findings? One possible explanation lies in the functional organization of the brain itself. Structural imaging captures anatomical change, yet neurological function depends largely on distributed neural networks rather than isolated regions. Consequently, radiation exposure affecting strategically interconnected pathways may disrupt functional communication long before overt structural injury becomes evident on conventional imaging.

This discrepancy highlights an essential limitation of interpreting post-radiotherapy MRI solely through morphological criteria. Imaging abnormalities should be understood not only as markers of tissue injury but also as indirect indicators of altered neuronal efficiency. In clinical practice, subtle expansion of FLAIR signal intensity may precede cognitive slowing, increased fatigue, or greater dependence on corticosteroid therapy, suggesting that imaging findings often represent early manifestations of functional imbalance rather than irreversible damage.

Another major challenge encountered during follow-up imaging involves differentiating tumor recurrence from treatment-related effects such as pseudoprogression or radiation necrosis. These entities share overlapping radiological characteristics because both arise from biologically active processes involving vascular permeability changes and inflammatory responses. From a physiological perspective, radiotherapy may be viewed as inducing a controlled injury intended to eradicate tumor cells, yet the surrounding brain tissue inevitably participates in the reparative response. Imaging therefore reflects not only destruction but also attempted recovery.

Importantly, radiation-related alterations frequently extend beyond regions receiving the highest radiation dose. Clinical experience increasingly suggests that areas exposed to intermediate dose levels may demonstrate diffuse microstructural changes contributing to neurological symptoms. This observation supports the concept that radiation-induced dysfunction may resemble network destabilization rather than focal tissue failure—an effect comparable to partial disruption within a communication system where overall performance declines despite preservation of individual components.

Advanced imaging modalities, including diffusion and perfusion MRI, have further refined the assessment of treatment-related injury by revealing microstructural and hemodynamic alterations preceding visible anatomical change. These techniques reinforce the notion that radiation-induced neuronal dysfunction represents a continuum rather than a binary event detectable only at advanced stages.

From a clinical perspective, these observations emphasize that treatment planning should consider not only target coverage but also preservation of functional brain regions exposed to intermediate radiation doses. Careful evaluation of dose distribution within healthy brain tissue may therefore contribute to reducing long-term neurological morbidity without compromising oncological outcomes.

**Figure 2:**
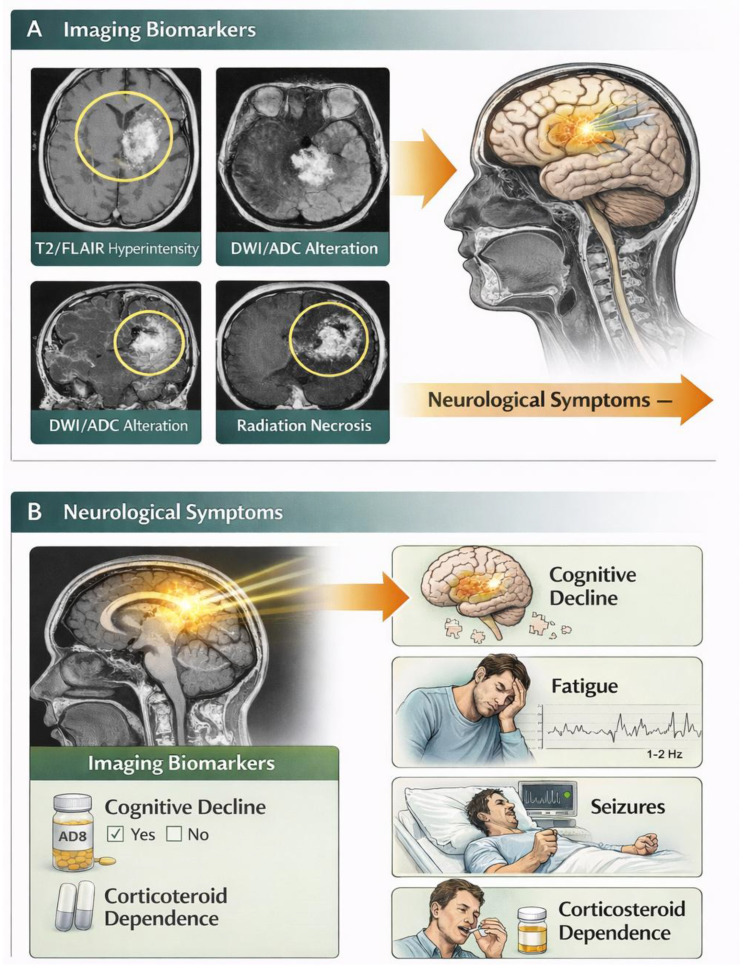
Schematic representation of imaging biomarkers and associated neurological manifestations following cranial radiotherapy. Illustrative examples demonstrate typical radiological patterns and related clinical symptoms. *Images are schematic illustrations and do not represent actual patient data.*

Beyond conventional MRI, advanced neuroimaging techniques have emerged as valuable tools for detecting early and subtle radiation-induced brain changes. Diffusion tensor imaging (DTI) allows the assessment of white matter integrity, revealing microstructural damage that may precede visible lesions on standard imaging. Functional MRI (fMRI) provides insight into alterations in brain network connectivity, demonstrating changes in functional organization even in the absence of overt structural abnormalities. Additionally, perfusion MRI techniques can identify vascular alterations and impaired cerebral blood flow, contributing to a more comprehensive understanding of radiation-induced injury. These advanced imaging modalities offer the potential for earlier detection, improved monitoring, and more precise correlation with clinical outcomes, as illustrated in Figure 2 [22,25,26].

## 6. Clinical Outcomes of Radiation-Induced Brain Injury

### 6.1. Cognitive Outcomes

Cognitive impairment represents one of the most clinically significant consequences of cranial radiotherapy [1,2]. Memory dysfunction, particularly involving hippocampal-dependent processes, is among the most frequently reported deficits, reflecting the high radiosensitivity of neural stem cell niches [5,6]. In addition to memory disturbances, impairments in attention, processing speed, and executive function have been described, suggesting that radiation-induced injury affects distributed neural networks rather than isolated brain regions [11,12].

These alterations may develop progressively and are often related to both direct neuronal damage and secondary mechanisms, including vascular injury, neuroinflammation, and disruption of synaptic connectivity. Importantly, even subtle cognitive deficits may have a significant impact on patient functioning and long-term outcomes.

### 6.2. Neurological Manifestations

Beyond cognitive decline, patients may experience a broad spectrum of neurological symptoms following radiotherapy. These include seizures, fatigue, and focal neurological deficits, which may arise from both direct radiation-induced injury and indirect mechanisms such as edema, blood–brain barrier disruption, and inflammatory processes.

In patients with brain tumors, these manifestations may be further influenced by tumor location, pre-existing neurological compromise, and concurrent treatments such as corticosteroids. The dynamic interplay between tumor-related and treatment-related effects makes clinical assessment particularly challenging.

### 6.3. Functional Impact and Quality of Life

The functional consequences of radiation-induced brain injury extend beyond measurable neurological deficits, significantly affecting quality of life and daily functioning [1,2]. Even mild cognitive impairment may translate into reduced independence, impaired social interaction, and decreased ability to tolerate oncological treatments [10,25]. 

From a broader perspective, radiation-induced brain injury can be understood as a disruption of functional brain networks rather than solely a localized structural lesion. This network-based dysfunction may explain the discrepancy often observed between imaging findings and clinical presentation, highlighting the need for more integrative assessment approaches.

## 7. Modern Radiotherapy Strategies and Neuroprotection

The evolution of modern radiotherapy has fundamentally transformed the therapeutic approach to brain tumors, shifting from uniform irradiation paradigms toward increasingly individualized treatment strategies. Historically, treatment planning primarily focused on achieving adequate tumor coverage, with normal brain tissue regarded largely as an unavoidable collateral exposure. Advances in imaging, planning algorithms, and delivery techniques have progressively challenged this perspective, introducing the concept that preservation of neurological function represents an equally relevant therapeutic endpoint.

Contemporary radiotherapy techniques such as intensity-modulated radiotherapy (IMRT) and volumetric modulated arc therapy (VMAT) allow highly conformal dose distributions capable of adapting radiation delivery to complex anatomical configurations. Beyond improving target coverage, these techniques enable meaningful reduction in radiation exposure to functionally critical brain regions. This technical refinement has introduced a conceptual transition in radiation oncology: treatment success is no longer defined solely by tumor control but increasingly by the balance between oncological efficacy and functional preservation.

An important consequence of this transition is the recognition that the brain cannot be considered a uniform radiosensitive organ. Distinct neural structures demonstrate varying susceptibility to radiation-induced injury depending on cellular composition, vascular characteristics, and functional specialization. The hippocampus represents one of the most extensively studied examples, where dose reduction strategies have demonstrated measurable benefits in memory preservation. However, emerging clinical observations suggest that functional vulnerability extends beyond isolated anatomical structures toward distributed neural networks responsible for cognition, attention, and behavioral regulation.

From a practical standpoint, neuroprotective radiotherapy increasingly relies on minimizing unnecessary irradiation of healthy brain tissue rather than attempting to eliminate radiation exposure entirely. Low- and intermediate-dose regions, once considered clinically negligible, are now recognized as potential contributors to long-term neurological dysfunction. Modern planning strategies therefore emphasize reduction in integral brain dose and optimization of dose gradients surrounding target volumes.

Another emerging consideration involves the dynamic nature of brain response during treatment. Radiotherapy planning traditionally assumes anatomical stability throughout treatment delivery; however, evolving edema, postoperative cavity changes, or treatment-related inflammation may alter spatial relationships between tumor and healthy tissue over time. Adaptive radiotherapy approaches aim to address these variations by modifying treatment plans according to interval imaging findings, thereby limiting unintended exposure of normal brain structures.

Importantly, neuroprotection should not be interpreted solely as a technical achievement but as a multidisciplinary objective. Pharmacological interventions, optimized corticosteroid management, seizure control, and supportive systemic therapies contribute significantly to maintaining neurological stability during and after treatment. In this broader framework, radiotherapy becomes one component within a coordinated strategy aimed at preserving functional independence.

A further conceptual shift concerns the integration of functional information into treatment planning. Advances in functional MRI and diffusion imaging have opened the possibility of identifying eloquent cortical regions and major white matter tracts prior to irradiation. Although still evolving, function-guided radiotherapy introduces the possibility of protecting neural connectivity pathways rather than exclusively anatomical landmarks. Such approaches acknowledge that neurological outcomes depend on preservation of communication between brain regions rather than survival of isolated structures alone.

An unresolved but increasingly relevant question arises from these developments: can radiotherapy itself become a tool for functional preservation rather than merely a source of potential injury? The answer may lie in the capacity of modern techniques to redistribute dose intelligently, allowing tumor eradication while maintaining sufficient neural reserve for adaptive recovery. In this sense, neuroprotective radiotherapy does not eliminate biological impact but seeks to maintain the brain’s ability to reorganize following treatment.

**Figure 3:**
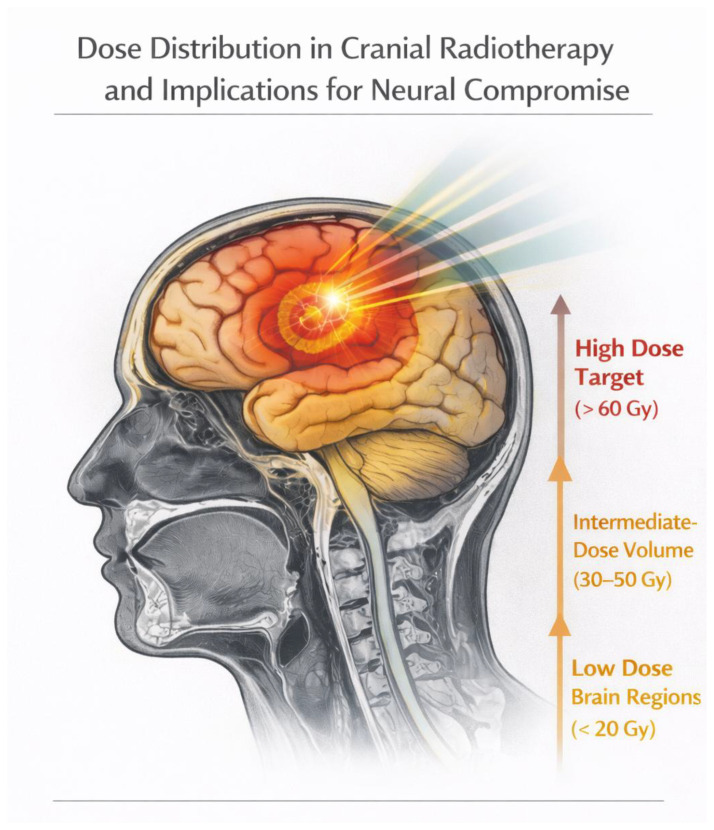
Schematic illustration of radiation dose distribution demonstrating high-, intermediate-, and low-dose exposure of healthy brain tissue during cranial radiotherapy. *Illustration created for educational purposes.*

Ultimately, modern radiotherapy reflects a transition from geometry-driven treatment toward biology- and function-oriented intervention. Continued refinement of dose optimization strategies, adaptive planning, and integration of functional imaging may progressively reduce treatment-related neurological morbidity. As survival improves, the success of cranial radiotherapy will increasingly be measured not only in months gained but in cognitive and functional capacity preserved throughout survivorship, as illustrated in Figure 3.

## 8. Future Perspectives

The growing recognition of radiation-induced neuronal dysfunction has prompted a gradual redefinition of therapeutic priorities in neuro-oncology. As survival outcomes continue to improve, future advances in cranial radiotherapy will likely focus not only on maximizing tumor control but also on preserving long-term neurological adaptability. This shift requires moving beyond traditional toxicity assessment toward predictive and preventive treatment strategies.

One emerging direction involves the transition from population-based dose constraints to individualized estimations of normal brain tolerance. Current planning approaches rely largely on generalized dose–volume recommendations derived from heterogeneous patient cohorts. However, clinical experience increasingly demonstrates substantial variability in neurological outcomes among patients receiving comparable radiation doses. Future treatment paradigms may therefore incorporate biological and clinical markers capable of identifying individual susceptibility to radiation-induced injury prior to treatment initiation.

Another promising development lies in the integration of longitudinal imaging biomarkers into treatment evaluation. Rather than interpreting post-treatment MRI changes retrospectively, future models may enable early detection of vulnerable brain regions during therapy itself. Continuous imaging assessment could allow adaptive modification of treatment plans, transforming radiotherapy into a dynamically responsive intervention rather than a fixed therapeutic course.

Artificial intelligence-based analysis represents an additional avenue with considerable potential. Machine learning algorithms applied to treatment planning data, imaging evolution, and clinical parameters may facilitate prediction of neurological toxicity before symptom onset. Such predictive frameworks could assist clinicians in balancing oncological benefit against functional risk, ultimately supporting personalized decision-making processes.

Beyond technological innovation, a conceptual transformation may also be required. Increasing evidence suggests that radiation-induced brain injury reflects progressive alteration of functional reserve rather than isolated structural damage. Future research may therefore focus on identifying strategies capable of maintaining neural resilience throughout treatment. In this context, radiotherapy planning could evolve toward preservation of functional connectivity networks, acknowledging that maintenance of communication between brain regions may be as important as protection of individual anatomical structures.

An important unanswered question remains whether early supportive interventions—pharmacological, metabolic, or rehabilitative—could mitigate functional decline when introduced proactively rather than reactively. Addressing radiation-induced neuronal dysfunction may thus require collaboration extending beyond radiation oncology, incorporating neurology, rehabilitation medicine, and cognitive sciences.

Ultimately, the future of cranial radiotherapy may depend on redefining treatment success. Prolonged survival alone may no longer represent the sole therapeutic objective; instead, preservation of cognitive autonomy and functional identity may emerge as equally meaningful outcomes. Advancing toward this goal will require integration of biological understanding, technological precision, and patient-centered clinical evaluation.

## 9. Limitations

This review represents a narrative synthesis of currently available literature and is therefore subject to limitations related to heterogeneity among published studies, variability in imaging protocols, and differences in reported neurological outcomes. Additionally, correlations between radiation dose distribution, imaging findings, and clinical manifestations remain influenced by patient-specific factors and treatment-related variability. Future prospective studies are required to further clarify these relationships.

## 10. Conclusions

Radiotherapy remains an indispensable component in the management of brain tumors, contributing substantially to disease control and patient survival. However, growing clinical experience has demonstrated that its impact extends beyond tumor eradication, influencing the structural and functional integrity of the surrounding healthy brain. Radiation-induced neuronal dysfunction should therefore be understood as a multifactorial and dynamically evolving process shaped by dose distribution, biological response, and individual patient vulnerability.

Accumulating evidence indicates that neurological outcomes following cranial irradiation cannot be explained solely by focal tissue injury. Instead, treatment-related effects frequently emerge from complex interactions involving neuroinflammation, vascular alteration, impaired neurogenesis, and disruption of functional neural networks. Imaging biomarkers and clinical manifestations represent complementary expressions of these underlying processes, emphasizing the need for integrated interpretation rather than isolated assessment.

Modern radiotherapy has progressively transitioned toward strategies aimed at minimizing exposure of functionally relevant brain regions while maintaining oncological efficacy. This evolution reflects a broader shift within neuro-oncology—from treating disease alone toward preserving neurological function and patient autonomy throughout survivorship.

Understanding radiation-induced brain injury as a disturbance of functional reserve rather than exclusively structural damage may provide a useful framework for future research and clinical decision-making. Such an approach encourages individualized treatment planning, early recognition of vulnerable patients, and multidisciplinary management focused on maintaining neurological resilience.

In this context, preservation of neurological function should be regarded as an essential therapeutic objective alongside tumor control, reflecting an evolving paradigm in modern radiation oncology.

Ultimately, the success of cranial radiotherapy may increasingly be defined not only by prolonged survival but by the preservation of cognitive capacity, functional independence, and quality of life. Continued integration of biological insight, advanced imaging, and personalized treatment strategies will be essential in achieving this balance between effective tumor control and protection of the human brain as the substrate of cognition and identity.

## Figures and Tables

**Table 1:** Dose–volume parameters and associated neurological outcomes reported in the literature.

Study	Dose–Volume Parameter	Brain Region	Reported Outcome
Gondi et al. [10]	Mean hippocampal dose	Hippocampus	Memory preservation with dose reduction
Lawrence et al. [3]	V20, mean brain dose	Whole brain	Increased risk of neurotoxicity with higher volume exposure
Makale et al. [2]	Dose-dependent effects	Multiple regions	Cognitive decline and network dysfunction
Greene-Schloesser et al. [1]	Radiation exposure	Hippocampus	Impaired neurogenesis and memory deficits
Parihar et al. [12]	Radiation dose	Hippocampus	Altered neuronal architecture and synaptic loss

## Data Availability

No new data were created or analyzed in this study. Data sharing is not applicable to this article.
